# Dimerisation of the Yeast K^+^ Translocation Protein Trk1 Depends on the K^+^ Concentration

**DOI:** 10.3390/ijms24010398

**Published:** 2022-12-26

**Authors:** Natalia Kulik, Deepika Kale, Karin Spurna, Katsiaryna Shamayeva, Fabian Hauser, Sandra Milic, Hannah Janout, Vasilina Zayats, Jaroslaw Jacak, Jost Ludwig

**Affiliations:** 1 Institute of Microbiology of the Czech Academy of Sciences, Zamek 136, 3733 Nove Hrady, Czech Republic; 2 School of Medical Engineering and Applied Social Sciences, University of Applied Sciences Upper Austria, Garnisonstr, 21, 4020 Linz, Austria; 3 Bioinformatics, University of Applied Sciences Upper Austria, 4232 Hagenberg, Austria; 4 Institute of Symbolic AI, Johannes Kepler University, 4040 Linz, Austria

**Keywords:** K^+^ translocation, *Saccharomyces cerevisiae*, bimolecular fluorescence complementation, dimerisation, molecular modelling, MD simulation

## Abstract

In baker’s yeast (*Saccharomyces cerevisiae*), Trk1, a member of the superfamily of K-transporters (SKT), is the main K^+^ uptake system under conditions when its concentration in the environment is low. Structurally, Trk1 is made up of four domains, each similar and homologous to a K-channel α subunit. Because most K-channels are proteins containing four channel-building α subunits, Trk1 could be functional as a monomer. However, related SKT proteins TrkH and KtrB were crystallised as dimers, and for Trk1, a tetrameric arrangement has been proposed based on molecular modelling. Here, based on Bimolecular Fluorescence Complementation experiments and single-molecule fluorescence microscopy combined with molecular modelling; we provide evidence that Trk1 can exist in the yeast plasma membrane as a monomer as well as a dimer. The association of monomers to dimers is regulated by the K^+^ concentration.

## 1. Introduction

**Figure 1:**
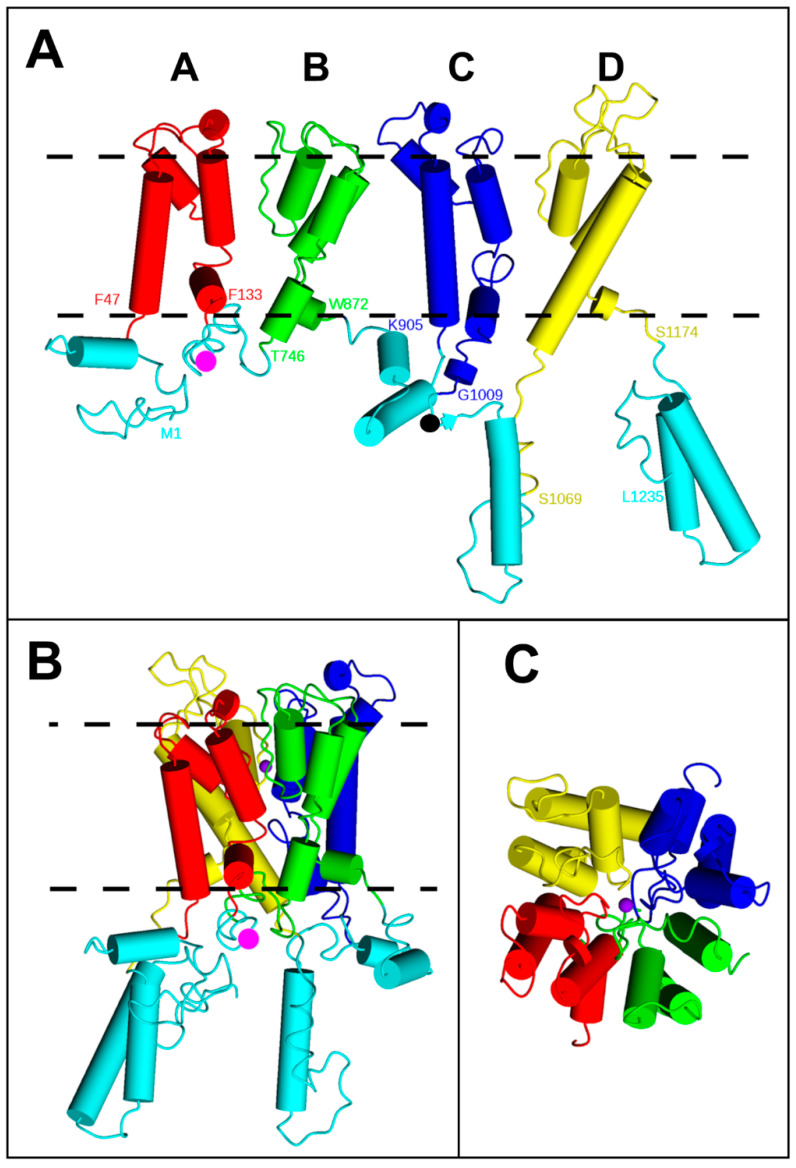
Structure of a Trk1[ΔLHL] monomer. (**A**) Schematic linear representation of a complete model of a Trkl monomer without the “Long Hydrophilic Loop” (LHL, residues 137–746) that is located between MPM domains A and B. (**B**,**C**) MPMs are arranged around a central pore; (**B**) side view; and (**C**) top view from the extracellular side. MPM domains are differently coloured: A: red, B: green, C: blue, and D: yellow. The same colour code is used in all the following figures. Terminal residues and residues at the membrane/cytosol borders are labelled with residue number in (**A**). N- and C-terminal stretches and intracellular loops are shown in cyan. Magenta dots in (**A**,**B**) indicate the position of LHL. The position in which GC was inserted into the third intracellular loop between MPMs C and D (G1010/GC/E1011, see results) is indicated by the black sphere in (**A**). The dashed lines in (**A**,**B**) indicate the plasma membrane. The violet sphere in (**C**) shows a potassium ion in the pore. N- and C-terminal regions as well as intracellular loops were modelled ab initio or using YASARA. For more details, see Section 4.

Trk1 is the main K^+^ uptake system in baker’s yeast (*Saccharomyces cerevisiae*). Deletion of *TRK1* prohibits growth in/on medium containing low K^+^ (see, e.g., [1,2]). Trk2 is a similar protein that, however, seems less important for promoting growth in limiting K^+^ media because its expression level is much lower [3]. In addition to their main role, the uptake of K^+^, Trk1, and Trk2 are involved in several other processes, such as the regulation of membrane potential [4,5]; and Ca^2+^ homeostasis [6]. Yeast Trks belong to the superfamily of K transporters (SKT), which is widespread in prokaryotes, fungi, and plants and consists of monovalent cation-translocation systems (for review, see, e.g., [7]). Most SKT proteins are thought to mediate passive ion translocation in the direction of the ions’ electrochemical gradient, but the family also includes a subunit of the K^+^ pump whose structure has been solved (KdpFABC; [8,9]). The cation-translocating parts of SKT proteins share the same principal architecture consisting of four Membrane-helix-Pore-Membrane helix (MPM) motifs (A–D) consisting of two transmembrane segments (M1 and M2) and a pore-forming region (P) between them (Figure 1A). Each MPM motif is thus homologouss to a 2-Transmembrane domain (2-TM) K-channel α-subunit. As K-channel α subunits the MPM motifs of Trks are arranged around a central pore (Figure 1B,C). It is worth mentioning that Stewart R. Durell and Robert H. Guy already proposed the similarity between SKT proteins and K-channels in 1999 [10]. They detected the evolutionary relationship of MPM motives between SKT-proteins families and K-channels [11] and developed structural models for different SKT proteins based on the then available crystal structure of the prokaryotic K channel KcsA [12] and multiple sequence alignment between SKT proteins and K-channels. Fifteen years later, when the crystal structures of the first SKT proteins (KtrB and TrkH) were solved, it became clear that many of their predictions were correct.

SKT proteins might contain auxiliary subunits like TrkA, which is associated with TrkH, and KtrA, which is associated with KtrB. Yeast Trk1 and Trk2 instead possess a so called “large hydrophilic loop” (LHL) that is not necessary for ion translocation but modulates the cation selectivity of Trk1 [13].

We previously developed an atomic scale model of Trk1 [14] based on the available crystal structures of TrkH [15] and KtrB [16] that are both dimers. Consequently, our model is also a dimer. However, the architecture of Trks, like that of the other SKT proteins, indicates that they could be fully functional as monomers. On the other hand, because the second TM helix of MPM D is highly charged and might be unstable in a hydrophobic environment, even a tetrameric organisation in which these charges would be hidden within a complex with MPM_D_ in the centre has been proposed from molecular modelling [10,11]. The presence of a central pore in the tetrameric arrangement was also proposed, which would allow for the observed anion permeability of Trk1 [17,18]. In order to elucidate the possible “multimeric” state(s) of Trk, we conducted a combined theoretical (molecular modelling and molecular dynamics (MD) simulations) and experimental study using bimolecular fluorescence complementation (BiFC) and single molecule fluorescence microscopy (SMFM).

## 2. Results

### 2.1. BiFC Indicates That Trk1[ΔLHL] Can Exist as a Dimer with an Interface Formed by MPMs C and D

**Figure 2:**
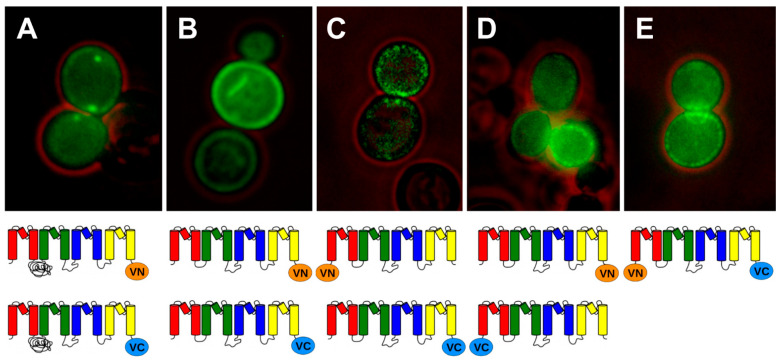
Trk1 monomers can interact, and LHL is not needed for association. (**A**) Trk1/VN + Trk1/VC; (**B**) Trk1[ΔLHL]/VN + Trk1[ΔLHL]/VC; (**C**) VN/Trk1[ΔLHL] + Trk1[ΔLHL]/VC; (**D**) VC/Trk1[ΔLHL] + Trk1[ΔLHL]/VN; and (**E**) VN/Trk1[ΔLHL]/VC. Below the pictures schemes of the (combined) Trk1([ΔLHL]) VC or VN fusions are shown.

As was shown previously using BiFC, Trk1 can form at least dimers (Kale et al., 2019). When the N- and C-terminal parts of the YFP variant Venus (VN and VC) were fused to the C-termini of Trk1 monomers (Trk1/VC and Trk1/VN) and co-produced in yeast, BiFC was observed (Figure 2A). Deletion of the long hydrophilic loop (LHL) did not affect fluorescence complementation, thus LHL is not required to form Trk1 dimers or multimers. On the contrary, co-expression of *TRK1[ΔLHL]/VN* with *TRK1[Δ LHL]/VC* led to increased BiFC fluorescence, most likely due to the removal of steric hindrance caused by LHL (Figure 2A,B).

Thus, all further experiments to elucidate the possible multimerisation of Trk1 were carried out with Trk1[ΔLHL]. First, BiFC was additionally tested with VN and VC fused to the N- and C-termini of either one monomer VN/Trk1[ΔLHL]/VC or two different monomers (VN/Trk1[ΔLHL] + Trk1[ΔLHL]/VC) and with VN and VC fused to the N-termini of two different monomers (VN/Trk1[ΔLHL] + VC/Trk1[ΔLHL]). The results showed that the N-and C-termini of one monomer (VN/Trk1[ΔLHL]/VC, Figure 2E) as well as of two different monomers were close enough to produce BiFC (VN/Trk1[ΔLHL] + Trk1[ΔLHL]/VC, Figure 2C,D), whereas the combination of VN/Trk1[ΔLHL] with VC/Trk1[ΔLHL] did not give rise to fluorescence (cf. Supplementary Figure S6). Thus, the N-termini of different monomers seemed to be too far from each other for BiFC.

**Figure 3:**
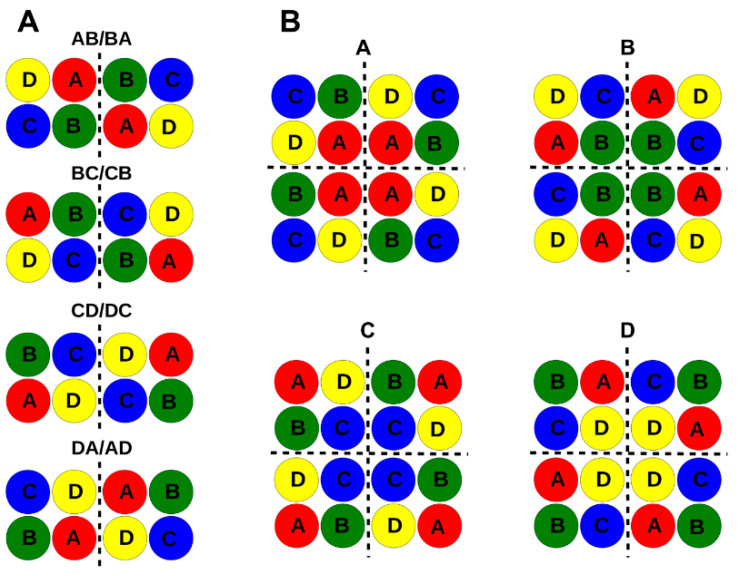
Possible arrangements of Trk1 monomers into symmetrical dimers and tetramers (top view). Each coloured circle represents the complete MPM motif indicated. (**A**) Dimers with interfaces between monomers formed by (from top to bottom) MPMs AB/BA, BC/CB, CD/DC, and DA/AD. (**B**) Tetramers in which a possible central pore could be formed by either MPM A, B, C, or D (colours as in Figure 1). The interfaces between monomers are indicated by dashed lines.

As mentioned above, the Trk1 polypeptide chain contains four MPM domains (A, B, C, and D), each homologous to a K-channel subunit, which are arranged around the central pore (Figure 1). If Trk1 monomers were to form symmetric dimers, the interfaces between the monomers could be formed by domains A-B/B-A, B-C/C-B, C-D/D-C, or D-A/A-D (Figure 3A). If functional Trk1 were symmetric tetramers, the interface between the monomers could be formed by each of the four domains, i.e., the monomers would be symmetrically arranged with these domains in the centre (tetramers A, B, C, and D, Figure 3B).

**Figure 4:**
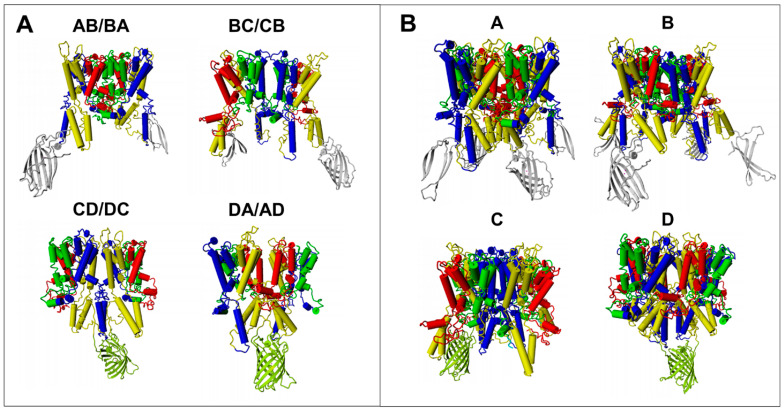
Models of possible arrangements of Trk1[ΔLHL] monomers with VN and VC attached to the C-termini of separate monomers. If VN and VC are close enough, the yellow/green colouring of the reconstituted fluorescent protein indicates a possible BiFC. (**A**) Symmetrical dimers with interfaces formed by the MPMs indicated on top. (**B**) Symmetric tetramers with the indicated MPMs in the centre. Theoretically, two fluorescent proteins could be reconstituted as tetramers. However, in most cases, this is prevented by clashes that occur between them.

Analysis of the electrostatic complementarity between the contact surfaces of dimers (Supplementary Figure S1) showed that the best complementarity was between the C-D and D-C surfaces. In addition, BC/CB and DA/AD dimers would be possible due to the presence of some complementary parts, whereas AB/BA interaction would be electrostatically unfavourable. All symmetric dimers and tetramers as well as a monomer were stable in the membrane in 100 ns MD simulations (Supplementary Figure S2). However, modelling and MD simulations did not allow for unambiguous differentiation between possible arrangements. Thus, atomic scale models based on a complete Trk1[ΔLHL] model (Figure 1) were generated in which VN and VC were attached to the N- and C-termini of Trk1[ΔLHL], respectively. The ability of fluorescence complementation for symmetric dimers and tetramers was then analysed in silico by modelling the respective arrangements and measuring the distance between VN and VC fragments on different Trk1[ΔLHL] monomers. If it seemed likely that VN and VC came within a suitable distance (less than ~50 nm), fluorescence complementation was predicted. Trk1[ΔLHL] was used for modelling and MD as in the “wet-lab” experiments because BiFC was stronger than with full length Trk1 (see above). Furthermore, there is no structural model of LHL available. In Figure 4, the models for Trk1[ΔLHL]/VC and Trk1[ΔLHL]/VN (cf. Figure 3) arranged as symmetric dimers or tetramers are shown.

According to these models, BiFC which was observed with Trk1[ΔLHL]/VN + Trk1[ΔLHL]/VC (Figure 2B), was consistent with dimers CD/DC, DA/AD, and tetramers with C and D in the centre. Modelling possible VN/Trk1[LHL] + Trk1[LHL]/VC assemblies revealed that the observed fluorescence complementation (Figure 2C) was possible for dimers CD/DC and DA/AD, as well as for tetramers B and C (Table 1 and Supplementary Figure S3C). VC/Trk1[LHL] + Trk1[LHL]/VN could lead to BiFC for dimers CD/DC and DA/AD, and all tetramers (Table 1 and Supplementary Figure S3E,F). BiFC was never observed when VN/Trk1[LHL] and VC/Trk1[LHL] were combined, which would have been expected if dimers AB/BA or BC/CB or tetramers A, B, or C were formed (Table 1, Supplementary Figure S3A,B). This combination could possibly also give rise to BiFC in the AD/DA dimer. However, the latter might be prevented by a potential sterical conflict between the loops connecting MPMs C and D. Taken together, the results left three possible arrangements of Trk1 monomers: CD/DC and DA/AD dimers, or a tetramer with four MPMs D in the centre (Table 1).

**Figure 5:**
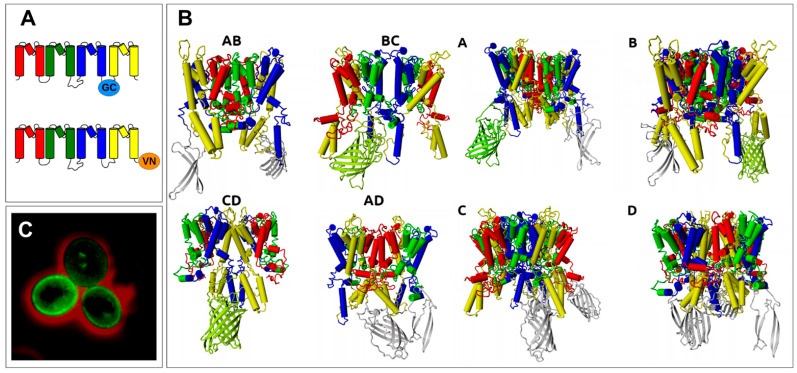
BiFC with GC inserted in loop three and VN attached to the C-terminus of Trk1[ΔLHL]. (**A**) Schematic representation of Trk1[ΔLHL] monomers with the C-terminal fragment of GFP (GC) inserted into intracellular loop three (Trk1[ΔLHL][G1010/GC/E1011], top) and VN fused to the C-terminus (Trk1[ΔLHL]/VN, bottom). (**B**) Models of symmetric dimers and tetramers indicating whether BiFC would be possible in the respective arrangement. (**C**) Fluorescence microscopy picture of (Trk1[ΔLHL][G1010/GC/E1011] plus Trk1[ΔLHL]/VN) cells.

Therefore, we looked for internal positions in Trk1[ΔLHL] at which the insertion of a fluorescent “half-protein” and analysis of BiFC with the other part attached to another monomer would allow distinguishing between the remaining monomer arrangement options. Best suited were possibly exposed regions in intracellular loops of Trk1 that, were on the one hand, close to the membrane and, on the other hand, restricted the positioning of the fluorescent half-proteins, allowing BiFC only with certain arrangements. It turned out that the insertion of the C-terminal part of the fluorescent protein (GC, the C-terminal fragment of GFP was used in this case, because BiFC was more easily detected by fluorescence microscopy visualisation with the setup used) into the intracellular loop three between MPM motifs C and D (Trk1[ΔLHL][G1010/GC/E1011]) in combination with Trk1[ΔLHL]/VN (Figure 5A) would allow BiFC only for BC/CB and CD/DC dimers and tetramers with A or B in the centre but not with dimers AB/BA and DA/AD and with tetramers C and D (Figure 5B). Therefore, the observed fluorescence complementation (Figure 5C) excluded the tetramer D and the DA/AD dimer, leaving only the CD/DC dimer.

### 2.2. Single Molecule Fluorescence Microscopy and Stepwise Photobleaching

**Figure 6:**
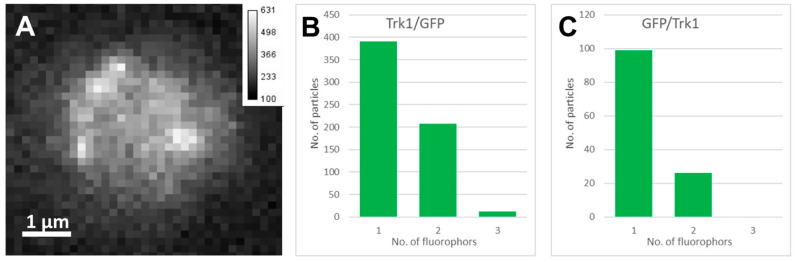
Stepwise photobleaching of Trk-GFP fusion proteins analysed by single molecule fluorescence microscopy (SMFM). (**A**) Fluorescent image of a Trk1/GFP cell. The scale bar gives the fluorescence intensity of particles (a.u., t_ill_ = 10 ms, P = 3.2 kW/cm^2^, average single-molecule signal intensity = 234.7 ± 47 cnts/pix). (**B**) Number of fluorophores in all fluorescent particles analysed in Trk1/GFP cells. (**C**) GFP/Trk1. In Supplementary Figure S5, a typical stepwise photobleaching trace of Trk1/GFP inside the cell is shown.

To test the proposed dimerisation, stepwise photobleaching (SP) analysis at the single-molecule level was performed. For these experiments, yeast cells were used, in which the *TRK1* gene was replaced with either *GFP/TRK1* or *TRK1/GFP* fusion genes. For SP analysis of the GFP signals 1000 frames with 10 ms illumination were captured (on average, a fluorescent GFP signal was observed in 223 ± 52 frames). Figure 6A displays a fluorescence image of a yeast cell with Trk1/GFP fluorescence signals. For SP analysis, sparsely distributed, diffraction-limited fluorescence signals (Figure 6A) were determined from a sequence of images that were analysed. A time trace of a bleaching sequence is shown in Supplementary Figure S5, where the intensity decay corresponds to the signal of two emitters within a diffraction-limited spot. The signal of a single emitter blinking can be seen later in the trace. The summarised SP results showed that Trk1/GFP (Figure 6B) and GFP/Trk1 (Figure 6C) GFP fusion proteins incorporated in the plasma membrane were monomers (corresponding to a single GFP emitter per diffraction-limited spot) and dimers (corresponding to two GFP emitters per diffraction-limited spot), with a majority of monomers (Trk1/GFP ~65%; GFP/Trk1 ~80%).

### 2.3. Quantification of BiFC Fluorescence

**Figure 7:**
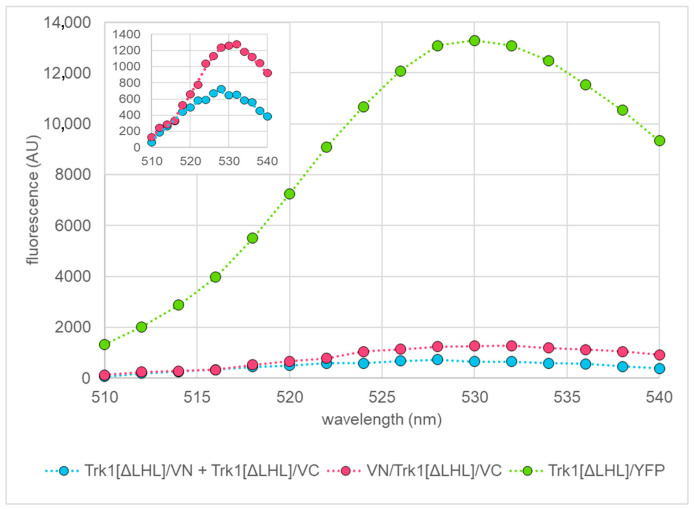
Quantification of BiFC. Representative BiFC fluorescence spectra of yeast cells grown in medium with 1 mM KCl and possessing the indicated Trk1[ΔLHL] fusion proteins. Spectra were recorded in the late exponential phase (OD ~2.5; growth time: 14 h). The spectra of Trk1[ΔLHL]/VN + Trk1[ΔLHL]/VC, and VN/Trk1[ΔLHL]/VC are magnified in the inset for better visibility.

To further analyse this finding, a semi-quantitative BiFC analysis was performed by recording BiFC-fluorescence emission spectra from cultures of yeast cells expressing *TRK1[ΔLHL]* fusion genes with VN and/or VC from high copy number plasmids in order to reach a sufficient signal-to-noise ratio. As positive control, Trk1[ΔLHL]/YFP cells were used. Recording emission spectra instead of measuring fluorescence only at one emission/excitation pair allowed assessing the quality of the measurements and rule out non-specific fluorescence or contaminations. For all measurements, the same number of cells (as determined by OD measurements) was used. The occurrence of BiFC when Trk1[ΔLHL]/VN and Trk1[ΔLHL]/VC were present indicated the presence of dimers, whereas BiFC with VN/Trk1[ΔLHL]/VC cells was taken as an estimate for monomers. It should be noted that if the N- and C-termini of different monomers interacted, VN/Trk1[ΔLHL]/VC could also result in BiFC. In initial “quantitative BiFC” experiments, it was found that the simultaneous presence of VN/Trk1[ΔLHL] and Trk1[ΔLHL]/VC led to much lower BiFC fluorescence as compared to the BiFC fluorescence observed with VN/Trk1[ΔLHL]/VC cells. However, we observed a slight increase of VN/Trk1[ΔLHL] + Trk1[ΔLHL]/VC BiFC signal in the late stationary phase in medium with 1 mM KCl. Thus, the dimer number might be somewhat underestimated. In initial experiments, it was also confirmed that VN and VC fused to the N-termini of separate Trk1[ΔLHL] monomers did not allow BiFC (Supplementary Figure S6). A set of such spectra in the exponential growth phase (12 h growth and ~2.75 × 10^7^ cells/mL in the original culture) in medium with 1 mM KCl is shown in Figure 7. As expected, Trk1[ΔLHL]/YFP exhibited by far the strongest fluorescence. Intramolecular BiFC (VN/Trk1[ΔLHL]/VC) was ~2 times as high as intermolecular BiFC (Trk1[ΔLHL]/VN + Trk1[ΔLHL]/VC cells), indicating a significant portion of monomers.

**Figure 8:**
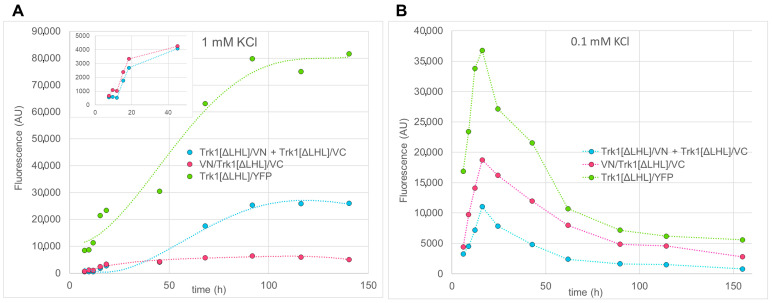
Time course of BiFC fluorescence for cells possessing Trk1 fusions with full or combinations of partial fluorescent proteins. (**A**) Cells grown in medium containing 1 mM KCl. For better visibility, exponential and early stationary phases of Trk1[ΔLHL]/VN plus Trk1[ΔLHL]/VC cells and VN/Trk1[ΔLHL]/VC cells are shown magnified in the insert. Dashed lines in the main panel: (phenomenological) fit of polynomials of 4th degree; inset: dashed straight lines connecting data points. (**B**) Cells grown in medium with 0.1 mM KCl. Data points are connected by straight dashed lines. Data shown are from single representative experiments.

Sets of such spectra were taken with media containing different concentrations of KCl during exponential and stationary growth phases. To quantify fluorescence as one data point per strain and measurement time, the average background-corrected “peak” fluorescence (530–534 nm) was taken. In Figure 8A the time course of BiFC fluorescence for VN/Trk1[ΔLHL]/VC and Trk1[ΔLHL]/VN + Trk1[ΔLHL]/VC cells in medium containing 1 mM KCl is shown. Fluorescence increased during the exponential growth phase and remained stable in the stationary phase throughout the time of the experiment. While in the exponential growth phase VN/Trk1[ΔLHL]/VC cells exhibited higher fluorescence, in the stationary phase, BiFC fluorescence with Trk1[ΔLHL]/VN + Trk1[ΔLHL]/VC is higher.

**Figure 9:**
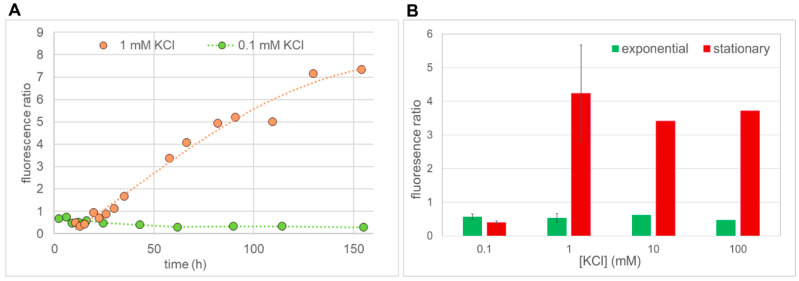
Ratios of BiFC fluorescence of cells expressing *TRK1[ΔLHL]*/*VN* plus *TRK1[ΔLHL]*/*VC* or VN/TRK1[ΔLHL]/VC. (**A**) Cells were grown in media with either 1 mM or 0.1 mM KCl, as indicated. Dashed lines: 1 mM KCl Phenomenological fit of a polynomial of 4th degree, 0.1 mM KCl: straight lines connecting data points (**B**) Average fluorescence ratios in the exponential (average of measurements between ~5 and ~25 h, green) and stationary growth phases (average of measurements between ~50 and 150 h, red) of cells grown in media supplemented with different [KCl]. The error bars for 0.1 mM KCl and 1 mM KCl represent the standard deviation of these averages from three independent experiments. Data for media with 10 mM and 100 mM KCl are from single-time course recordings.

Similar patterns were observed when cells were grown in 10 mM and 100 mM KCl (not shown). Additionally, under these conditions, the VN/Trk1[ΔLHL]/VC BiFC signal was higher than the Trk1[ΔLHL]/VN + Trk1[ΔLHL]/VC BiFC signal in the exponential growth phase and lower in the stationary phase, indicating an increasing fraction of dimers. In low [KCl] (0.1 mM; Figure 8B), the YFP and BiFC fluorescence also increased during the exponential growth phase but decreased after reaching a peak in the late exponential phase. Under these conditions, the intermolecular BiFC fluorescence observed with Trk1[ΔLHL]/VN + Trk1[ΔLHL]/VC was lower than the BiFC fluorescence observed with VN/Trk1[ΔLHL]/VC throughout the whole experiment. In Figure 9A, the ratios of Trk1[ΔLHL]/VN + Trk1[ΔLHL]/VC fluorescence to VN/Trk1[ΔLHL]/VC as an estimate for the relative abundance of dimers and monomers in low (0.1 mM) and moderate (1 mM) KCl-containing media are plotted vs. growth time. Whereas in medium containing 0.1 mM KCl, this ratio is smaller than one and rather constant during the full time of the experiment, in medium containing 1 mM KCl, this ratio strongly increased during stationary phase, indicating an increase in dimers. The results of these experiments are summarised in Figure 9B, in which the average ratios in the exponential growth phase (from ~5 to ~25 h) and stationary growth phase (from ~50 to ~150 h) are shown. The data confirmed that, regardless of the KCl concentration, intramolecular BiFC fluorescence (VN/Trk1[ΔLHL]/VC), indicating monomers, seemed to be predominant in exponential growth phase. However, with higher [KCl], when the availability of K^+^ is not growth limiting, dimerization occurs during the stationary phase.

## 3. Discussion

The primary aim of this study was to find out whether yeast Trk1 exists in the plasma membrane in monomeric, dimeric, or even in a higher multimeric form. Trk1 could be fully functional as a monomer because its structure is homologous to a concatemer of four K-channel α-subunits and thus equivalent to a full K-channel. However, the related SKT proteins KtrB and TrkH, which work as K-channels, have been crystallised as dimers [15,16], and for Trks, a tetrameric structure has been proposed by earlier modelling [10].

We addressed this question by using BiFC with fusion proteins carrying a partial fluorescent protein (VN or VC) fused either to the N- or the C-terminus of Trk1[ΔLHL] monomers and co-expressing combinations of these fusion genes. It turned out that (i) the C-termini of Trk1 and the version lacking the long hydrophilic loop (LHL) and (ii) the N- and C-termini of separate monomers are close enough to give rise to BiFC (Figure 2), whereas BiFC was never observed (measured) when the parts of the fluorescent proteins were fused to the N-termini of separate Trk1 or Trk1[ΔLHL] monomers. The latter finding did not only show that the N-termini of Trk1 monomers were too far away for BiFC but also proved that the observed BiFC was not due to non-specific interaction/aggregation. Thus, Trk1 monomers can at least form dimers. However, the presence of such dimers does not exclude the additional presence of monomers or higher-order multimers (tetramers). It also does not tell which parts (MPMs) of Trk1 form the interface between the monomers. The possible (and most likely) arrangement would be symmetrical dimers or tetramers. Dimer interfaces could be formed by MPMs AB/BA, BC/CB, CD/DC, or DA/AD. Tetramers would be arranged with one of the four MPMs in the centre (Figure 3). Using molecular modelling and MD simulation, it was analysed which of the possible arrangements were consistent with the BiFC results that showed close proximity of the C-termini as well as the N- and C-termini of separate monomers but not of the N-termini (Figure 2). It turned out that these results were consistent with a tetramer around MPMs D and dimers CD/DC and DA/AD (Table 1). The insertion of a fluorescent protein fragment (GC) into Trk1[ΔLHL] intracellular loop three, connecting MPMs C and D (Trk1[ΔLHL][G1010/GC/E1011]) and the co-expression of the fusion gene with TRK1[ΔLHL]/VN, allowed to exclude the D-centred tetramer and the AD/DA dimer. Thus, the non-monomeric form of Trk1 is most likely a dimer, with the interface formed by MPMs C and D. This is consistent with the crystal structures of TrkH and KtrB, which also consist of dimers with a CD/DC interface.

Trk1 can also mediate anion translocation [17], and it was proposed that the permeation path was formed by the central interface of a D-tetramer [18]. Our results argue against this hypothesis and indicate that the anion-selective “pore” of Trk1 is formed either by the CD interface or even within one monomer. During MD simulations, an increased density of Cl^−^ ions was observed close to the intracellular pore exit (Supplementary Figure S4). This could be explained by the presence of positively charged residues (Arg and Lys) in this region or simply by interactions with K^+^ ions in the pore region. However, Cl^−^ ions were attracted to this region even in the absence of K^+^. Additionally, an increase in the density of Cl^−^ was seen close to the intracellular part of the CD interface. However, the simulations never showed the formation of a water-filled cavity or pore in the CD/DC contact surface that would allow Cl^−^ translocation. One might consider the possibility of a dimer of CD/DC dimers. However, this seems unlikely because in that case an interaction of the N-termini of Trk1[ΔLHL] (in VN/Trk1[ΔLHL] + VC/Trk1[Δ LHL]) located in different dimers would be possible. Thus, the Cl^−^ path via Trk1 remains elusive.

The fact that Trk1 as observed can exist as a dimer but not as a tetramer in the plasma membrane does not exclude the existence of monomers. Strictly speaking, it also does not exclude the existence of trimers or even higher-order multimers. However, this has never been observed for any K-channel-related protein (i.e., possessing four MPM motifs) and thus seems very unlikely. Therefore, single molecule fluorescence microscopy (SMFM) was used as an independent method. For these experiments, yeast cells were used in which the Trk1 gene was replaced with a TRK1/GFP or a GFP/TRK1 fusion gene. It turned out that, as expected, often two GFP molecules were in close proximity, indicating dimers. However, the majority of Trk1/GFP and GFP/Trk1 fluorescence was caused by single GFP molecules, showing the presence of monomers (Figure 6). In addition, very few fluorescent particles consisting of three fluorophores were observed that might have been caused by non-specific aggregation.

In order to analyse the distribution of Trk1 monomers and dimers under various conditions, “semi quantitative BiFC” analysis was performed. As indicators for dimers cells possessing Trk1[ΔLHL]/VN and Trk1[ΔLHL]/VC were used, and as a measure for monomers VN/Trk1[ΔLHL]/VC cells were employed. The dimerisation of the latter could also lead to BiFC via N-terminus–C-terminus interactions between two separate monomers. However, BiFC in cells with Trk1[ΔLHL]/VC plus VN/Trk1[ΔLHL] was very low as compared to those with VN/Trk1[ΔLHL]/VC, leading only to a small overestimation of the number of monomers. The results (Figure 8) showed that in medium with moderate and high [KCl] (≥1 mM), BiFC fluorescence (and YFP fluorescence in Trk1[ΔLHL]/YFP) increased during the exponential and early stationary growth phases and remained constant for a long period (Figure 8A). In contrast, in medium with low KCl (0.1 mM), YFP and BiFC fluorescence reached a maximum in the late exponential growth phase and decreased again during the stationary phase (Figure 8B).

It has been shown that yeast cells grown in media with non-limiting [K^+^] also contain higher internal [K] (see, e.g., [19]). Thus, a reason for this difference could be the increased stability of Trk1 in cells containing higher K^+^ concentrations. Alternatively, the metallothionine promoter P_CUP1_ that drove the expression of the *TRK1[ΔLHL]* fusion genes might be more active in the intracellular conditions present when cells are grown in higher [K^+^] media. It also seems possible that the Trk1 dimers are protected against degradation/recycling, i.e., dimerisation could stabilise the complex in the membrane by “hiding” regions sensitive to proteolysis in the inner part of the complex, making them less accessible. Experiments with Trk1 concatemers, for example, could be carried out to differentiate between these possibilities. However, since the analysis of this observation was not the main topic of the study, this was not followed further.

Importantly, the time course of the ratio of BiFC fluorescence of Trk1[ΔLHL]/VN plus Trk1[ΔLHL]/VC and VN/Trk1[ΔLHL]/VC cells differed strongly between these [K^+^]. In the exponential phase VN/Trk1[ΔLHL]/VC BiFC fluorescence indicating monomers was always higher than the dimer indicating BiFC fluorescence of Trk1[ΔLHL]/VN plus Trk1[ΔLHL]/VC cells. However, in the stationary phase, BiFC fluorescence of Trk1[ΔLHL]/VN plus Trk1[ΔLHL]/VC cells was strongly increased in moderate and high [KCl]-containing media, indicating that the major fraction of Trk1[ΔLHL] consisted of dimers (Figure 9). This is also consistent with the observation that the VN/Trk1[ΔLHL] plus Trk1[ΔLHL]/VC BiFC signal seemed to be increased in comparison to the VN/Trk1[ΔLHL]/VC signal in the late stationary phase in a 1 mM KCl-containing medium.

An unusual feature of Trk1 is that this translocation system can change its affinity from high to low depending on K^+^ [20,21,22]. It was thought that this change in affinity was caused by some molecular switch dependent on intracellular [K^+^]. In a recent study, it was claimed that not only intracellular but also extracellular K^+^ contributed to the affinity change and that affinity changes gradually [23].

Most microorganisms employ different high- and low-affinity membrane transport systems in order to cope with the changing availability of nutrients. A prominent example in yeast is glucose uptake. Here a wide variety of transporters with different affinities and transport capacities exist, and their expression is regulated according to the glucose availability determined intracellularly and via transmembrane sensors (for review, see, e.g., [24,25]. It is much less common that one transport protein changes its affinity in order to fulfil the transport requirements. Among them is a dual-affinity glucose uptake system, Igt1, that has recently been identified in *Torulaspora delbrueckii* [26]. AtKUP1 [27,28] from *Arabidopsis thaliana* is the only K^+^ translocation system besides Trk1 that was proposed to be able to switch between high and low affinity modes dependent on the extracellular K^+^ concentration. However, the mechanism of affinity change is still unknown. The best understood dual-affinity transporter is NRT1.1/CHL1 [29], which mediates nitrate transport in plants. The current model for NRT1.1 affinity shift is that in the presence of high NO^3−^ concentrations, NRT1.1 is present as a dimer that mediates nitrate translocation with low affinity (Kd ~2 mM). The switch of NRT1.1 to high affinity (Kd ~100 µM) is caused by the phosphorylation of a single threonine residue. This in turn leads to a de-dimerisation, and both monomers then act as high-affinity NO^3−^ transporters [30]. It is tempting to speculate that the affinity change in Trk1 might be caused by a similar/analogous mechanism. The results presented here show that Trk1 dimers are the dominant form in high [K^+^] in the stationary growth phase when no high velocity/high affinity uptake is needed. In the exponential growth phase and in the stationary phase, when [K^+^] is low and high K^+^ uptake is needed for growth, however, the fraction of monomers is dominant. MD simulations suggest that the pore of a single Trk1 monomer is more symmetrically built than the two pores in a dimer. This increased symmetry might lead to better coordination of the translocated ions and thus higher fluxes. At present, this explanation is still somewhat speculative. It remains to be determined in further studies whether the dimer-monomer ratio of Trk1 observed here is correlated to the K^+^-translocation capabilities of the protein.

## 4. Material and Methods

### 4.1. Strains and Growth Conditions

Most *Saccharomyces cerevisiae* strains used in this study were generated by (co-) transformation of BY4741 [31] *trk1*, *trk2*, and *tok1Δ* (=BYT123 in [32]) with plasmids containing *TRK1([ΔLHL])* fusion constructs with the N-terminal fragment consisting of residues 1–155 (VN) or the C-terminal fragment 156–238 (VC) from the GFP variant “Venus” [33]. With the setup we used, bleaching of VN/GC BiFC fluorescence was subjectively reduced compared to VN/VC fluorescence and thus more easily detected by fluorescence microscopy visualisation. Therefore, the C-terminal fragment (residues 155–238) of yEGFP (GC) was used instead of VC for the construction of pYEX-[HIS3]-*TRK1[ΔLHL][G1010/GC/E1011]*, in which the BiFC-fragment was inserted in intracellular loop three, connecting MPMs C and D of Trk1 (cf. Figure 1). In all cases, high copy number plasmids (derived from pYEX; cf. Supplementary Table S1) were used to obtain a sufficient signal-to-noise ratio.

Yeast strains Trk1/GFP (BY47141 *trk1*::*TRK1/GFP-loxP*) and GFP/Trk1 (BY47141 *trk1*::*loxP-Trk1/GFP*) were generated using a modification of the Cre-loxP disruption system [34]. For BY4741 *trk1*::*TRK1/GFP-loxP* nucleotides 3361–4204 of the *TRK1* gene *(CDS: 3361–3708 3′-non coding: 3709–4204)* of BY4741 were first replaced with *TRK1* (nucleotides 3361–3705)/*GFP* (nucleotides 4–717, encoding residues 2–238)/*TRK1* (STOP, nucleotides 3706–3708)/*loxP*/*HIS3*/*loxP*/*TRK1* (3′-non coding region, nucleotides 3709–4304) obtained from pUG6(HIS) TRK1->TRK1/GFP by homologous recombination and selection for histidine auxotrophy. Subsequently, cells were transformed with the Cre-recombinase expression plasmid pSH47 that carries the *URA3* gene as a selectable marker in order to remove the *HIS3* gene from the genome. pSH47 loss was obtained by streaking cells on 5′-FOA plates to counter-select for URA3 auxotrophy. *GFP/TRK1* was constructed analogously by initially using a homologous recombination replacement cassette containing *TRK1* (nucleotides −500–−1)/*loxP*/*HIS3*/*loxP*/linker (taggtgatatcagatccactagtccacc)/*GFP* (nucleotides 1–717, encoding residues 1–238)/*TRK1* (nucleotides 1–563). A list of strains used in this study is given in Supplementary Table S2.

Cells were grown as described previously [13] aerobically at 28 °C with rotational shaking at 180 rpm in Synthetic Dextrose Arginine Phosphate (SDAP) liquid medium [22], supplemented with the appropriate amino acids and the desired amount of KCl.

### 4.2. Plasmid Construction and Yeast Transformation

*E. coli*/*S. cerevisiae* shuttle plasmids derived from pYEX-BX (Clontech Laboratories, Mountain View, CA, USA). For co-expression experiments, pYEX-[*LEU2*] was used in combination with pYEX-[*HIS3*] [13]. All constructs carrying fusion genes of *TRK1*[ΔLHL] YFP or BiFC fragments were generated by PCR using the overlap extension method [35]. A list of all plasmids is given in Supplementary Table S1. All PCR-derived parts of plasmids were verified by sequencing. Maps and full sequences of all plasmids used in this study are available from the corresponding author. Episomal plasmids used for producing Trk1([ΔLHL]) fusion proteins are listed in Supplementary Table S1. For transformation, the LiCl/PEG method [36] was used.

### 4.3. Fluorescence Microscopy

Fluorescence microscopy was carried out as described in [37] using an epifluorescence microscope with a 100×/1.30 oil immersion lens and appropriate filter sets. Pictures were taken with a CCD camera. To distinguish YFP, VN/VC (Venus), and VN/GC fluorescence from non-specific fluorescence, two filter sets were used: λ_ex_: 450–490 nm, beam splitter 505 nm, λ_em_: 520 nm long pass for “specific” fluorescence, and λ_ex_: 510–560 nm, beam splitter 575 nm, λ_em_: 590 nm long pass for “non-specific” fluorescence. The fluorescent microscopy pictures shown were generated by subtracting non-specific fluorescence (λ_ex_ 510–560 nm) from pictures taken with λ_ex_ 450–490 nm and merging the result (green channel) with the corresponding bright field picture (red channel) using ImageJ [38].

### 4.4. Single Molecule Fluorescence Microscopy (SMFM)

For the experiments, yeast cells expressing *TRK1/GFP* or *GFP/TRK1* were grown in medium supplemented with 0.1 mM KCl and used for measurements in the late exponential growth phase. For the measurements, a suspension of living yeast cells was transferred into a reservoir prepared with a two-component adhesive on a glass cover slip. To immobilise the cells, the cover slip surface was coated with poly-D-lysine (1 mg/mL; incubation time: 10 min).

Image acquisition was performed as described previously [39,40] using a modified Olympus IX81 inverted epifluorescence microscope with an oil-immersion objective (UApo N 60×/1.49 NA, Olympus, Vienna, Austria). The sample was positioned on a XYZ piezo stage (P-733.3DD, Physical Instruments, Karlsruhe, Germany) with nanometer precision on top of a mechanical stage with a range of 1 × 1 cm. The sample was illuminated in the blue channel with 488 nm laser light from a diode laser (Toptica Photonics, Gräfelfing, Germany). The signal was detected using an Andor iXonEM+ 897 (back-illuminated) EMCCD camera (16 µm pixel size). The following filter sets were used: dichroic filter (ZT405/488/561/640rpc, Chroma, Olching, Germany), emission filter (446/523/600/677 nm BrightLine quad-band band-pass filter, Semrock, Rochester, NY, USA). Cell segmentation [41], localisation and stepwise photobleaching of GFPs were analysed using a software platform developed in-house. A detailed protocol can be found in the supplemental protocol “Analysis of stepwise photobleaching of GFP in cells”.

### 4.5. Quantification of BiFC

Yeast cell cultures were inoculated to an OD600 = 0.1 (~1.1 × 10^6^ cells/mL) and grown in SDAP medium with the required supplements and the appropriate amount of KCl at 28 °C, shaking at 180 rpm. For measurements, samples corresponding to 2.4 OD units were harvested by centrifugation (4000× *g*, 10 min, RT) and resuspended in 200 µL of water. Samples were transferred to 96-well microplates and after 2 h of settling to the bottom of the wells, emission spectra (λ_ex_ 474 nm, bottom, bandwidth 12 nm) from 510 to 540 nm (bandwidth 5 nm) were recorded in 2 nm steps using a fluorescence reader (Safire, Tecan, Groedig, Austria). To correct for non-specific fluorescence, spectra from cells not possessing a fluorescent Trk1-protein (Trk1[ΔLHL]/VC-L or Trk1[ΔLHL]/VN-L) were subtracted.

### 4.6. Molecular Modelling and MD Simulations

Dimer and tetramer models of Trk1 were built from the published homology model of the Trk1 monomer (Zayats et al., 2015) in YASARA (Krieger, Koraimann, and Vriend, 2002) based on the crystal structure of the KtrB dimer (pdb 4j7c) [16] taking into account the position of the crystal structure in the membrane as predicted by the OPM server. Local clashes on contact surfaces were resolved by local energy minimisation in vacuum using YASARA with a Nova force field [42]. The potential energy of modelled dimeric and tetrameric complexes was minimised and equilibrated by MD. Complexes of Trk1 structures embedded in a POPC bilayer membrane and solvated in water (the TIP3P model) were built with the CHARMM-GUI server (https://charmm-gui.org/ (accessed on 1 February 2022)). MD simulations were run for all complexes for 100 ns with GROMACS 4.6.5 using the following MD parameters: NPT ensemble, temperature set to 310 K and controlled by a Nose–Hoover thermostat; constant pressure at X-Y direction was controlled by a Parrinello–Rahman barostat and a CHARMM36m force field [43]. The stability of complexes was analysed by RMSD calculations (Supplementary Figure S2). Electrostatic surface potentials were calculated with YASARA by a numeric method and visualized using atom radii for surface representation. The maximum electrostatic surface potential for the colour range is 80 kcal/mol. The “Particle Mesh Ewald Approach” was used to calculate electrostatic potential in vacuo [44]. Protein-protein docking based on shape complementarity was carried out using Patchdock [45]. BLAST was used to search for homologs [46]. Secondary structure predictions were performed by Jpred [47]. Ab initio modelling was run on the I-TASSER server [48]. YASARA geometry-based loop modelling [49] was used for modelling of N- and C-terminal “tails” and intracellular loops and re-sampling. Structure alignments were carried out using the MUSCLE method in YASARA [50].

## Figures and Tables

**Table 1:** From modelling expected (possible) BiFC and observed BiFC with different N- and C-terminal Trk1[ΔLHL] fusions with VN and VC.

	VC/Trk1[ΔLHL]	VC/Trk1[ΔLHL]	Trk1[ΔLHL]/VC	Trk1[ΔLHL]/VN	
	VN/Trk1[ΔLHL]	Trk1[ΔLHL]/VN	VN/Trk1[ΔLHL]	Trk1[ΔLHL]/VC	
Result	NO BiFC	BiFC	BiFC	BiFC	Consistent
Possible arrangement					
Dimer AB/BA	no	no	no	no	no
Dimer BC/CB	yes	no	no	no	no
Dimer CD/DC	**yes**	**yes**	**yes**	**yes**	**yes**
Dimer DA/AD	**yes**	**yes**	**yes**	**yes**	**yes**
Tetramer A	yes	no	yes	no	no
Tetramer B	no	yes	yes	no	no
Tetramer C	no	yes	yes	yes	no
Tetramer D	**yes**	**yes**	**yes**	**yes**	**yes**

BiFC experiments with VN and VC attached to the N- or C-terminus are consistent with dimers CD/DC, DA/AD, and tetramer D.

## Data Availability

The data presented in this study are presented in the article and the supplementary material. Molecular cloning data (plasmid maps and full sequences) are available from the authors upon request.

## Supplementary Materials

The supporting information can be downloaded at: https://www.mdpi.com/article/10.3390/ijms24010398/s1.
