# Supplementary material for: Dimerisation of the Yeast K+ Translocation Protein Trk1 Depends on the K+ Concentration
Source: Int J Mol Sci. 2022 Dec 26;24(1):398. doi: 10.3390/ijms24010398 (PMC9820094; doi:10.3390/ijms24010398)
Supplement: Supplementary file 1 [file ijms-24-00398-s001.zip › ijms-2076561-supplementary.pdf]

Dimerization of the yeast K^+ translocation protein Trk1 depends on the K^+-concentration

Supplementary Material

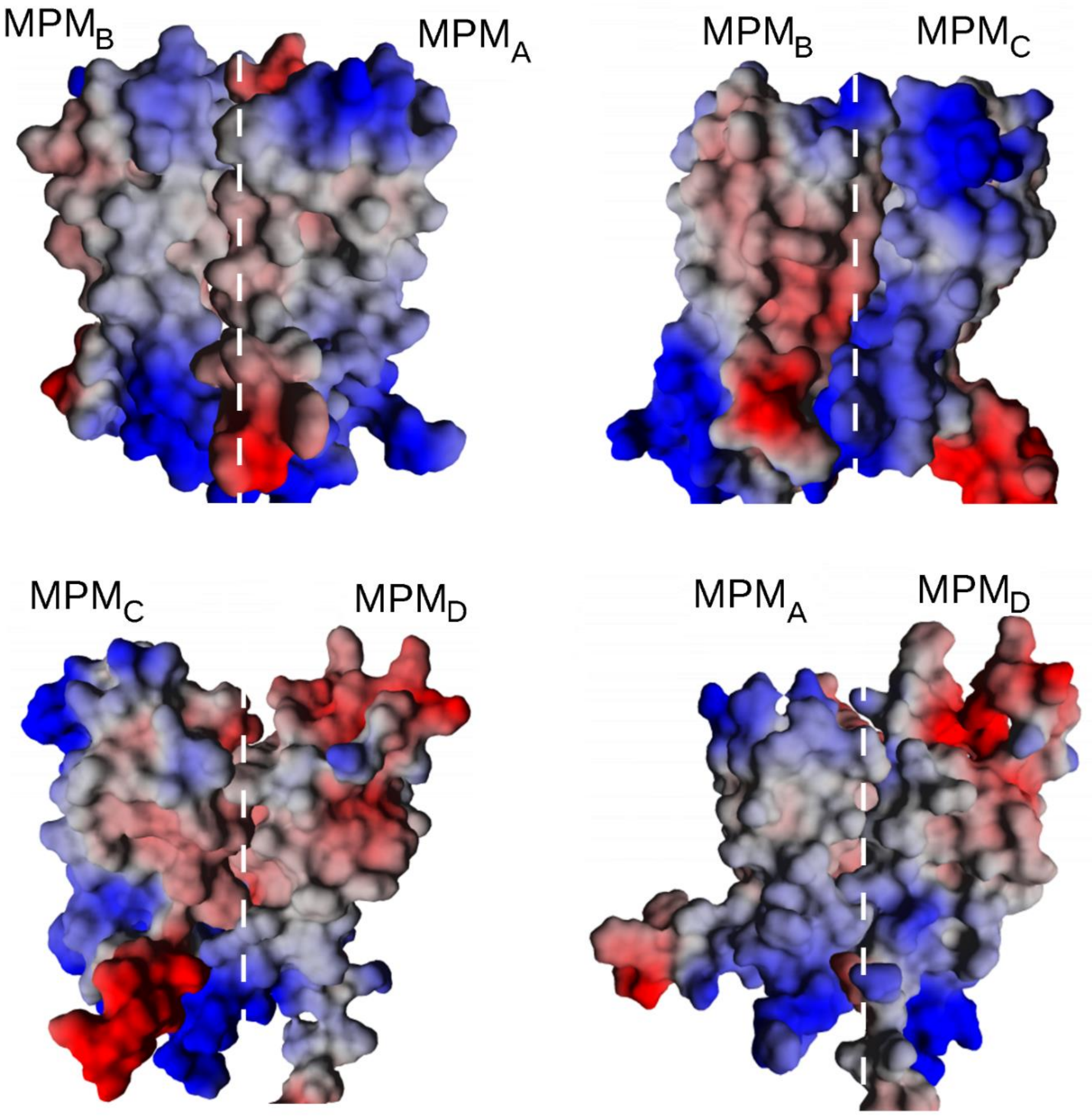

Supplementary Figure S1: Monomer contact surfaces of Trk1[ΔLHL] dimers coloured according electrostatic surface potential calculated by Particle Mesh Ewald method (Krieger *et al.*, 2006). The dashed line indicates the symmetry plane. MPMs, which participate in surface formation, are indicated. Positively charged residues are shown in red, and negatively charged residues are in blue. MPM A and MPM B possess negatively charged parts at the bottom of the interface, which will repel each other. In a possible BC/CB dimer MPM B has a positive and MPM C a negatively charged contact surface which may lead to favourable electrostatic interaction. Similarly, MPM C and MPM D have complementary charged contact surfaces. In an AD/DA dimer the upper parts of the monomer contact regions are oppositely charged while at the middle part charge distribution is similar. The bottom part of the AD/DA contact surfaces are both negatively charged and their interaction at this region is thus energetically not favourable.

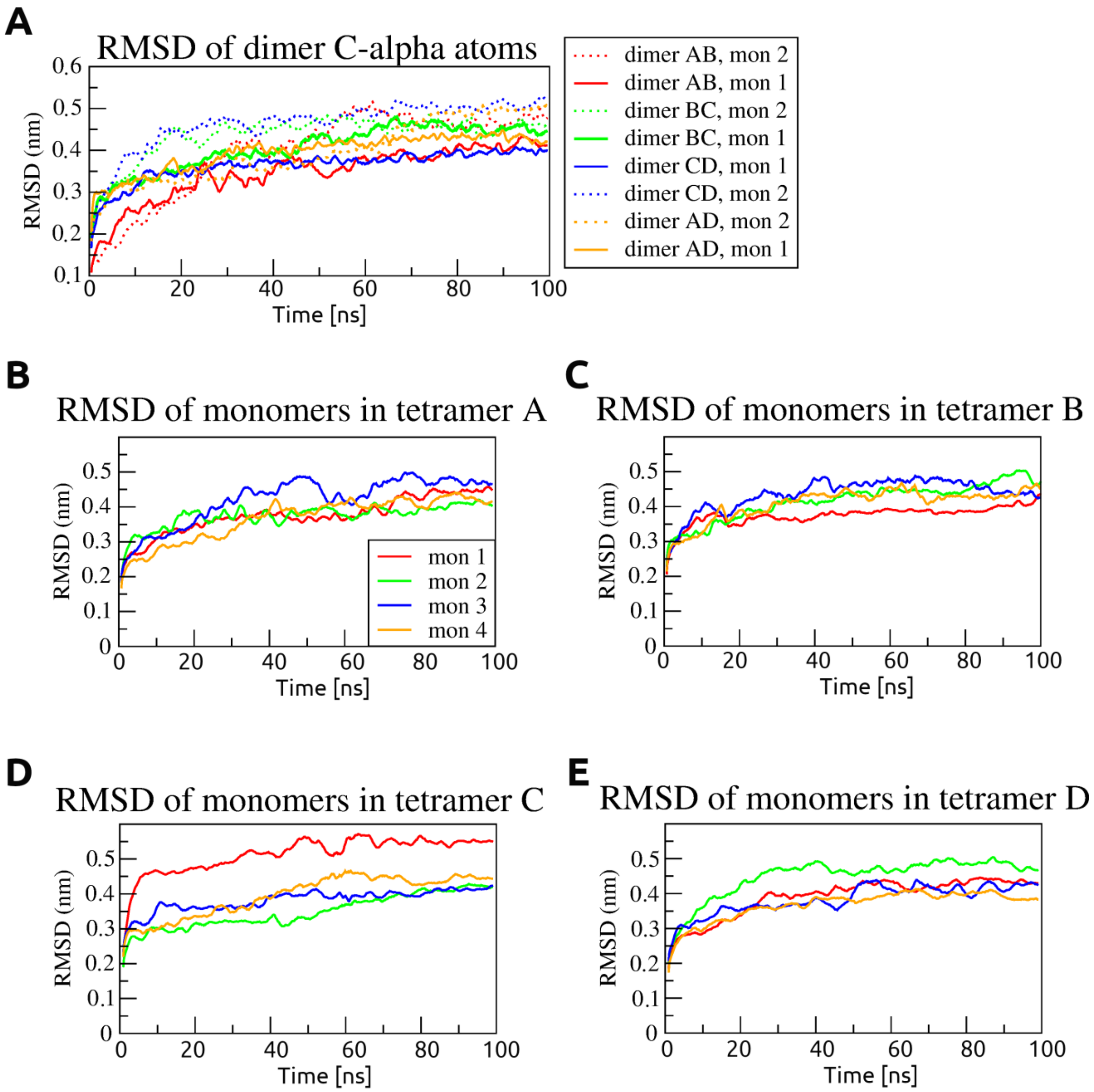

Supplementary Figure S2. RMSD of C-α atoms of monomers in different assemblies of Trk1[ΔLHL] monomers during 100 ns MD simulation. A: symmetrical dimers, B-E: Tetramers. Data are averaged over 10 snapshots.

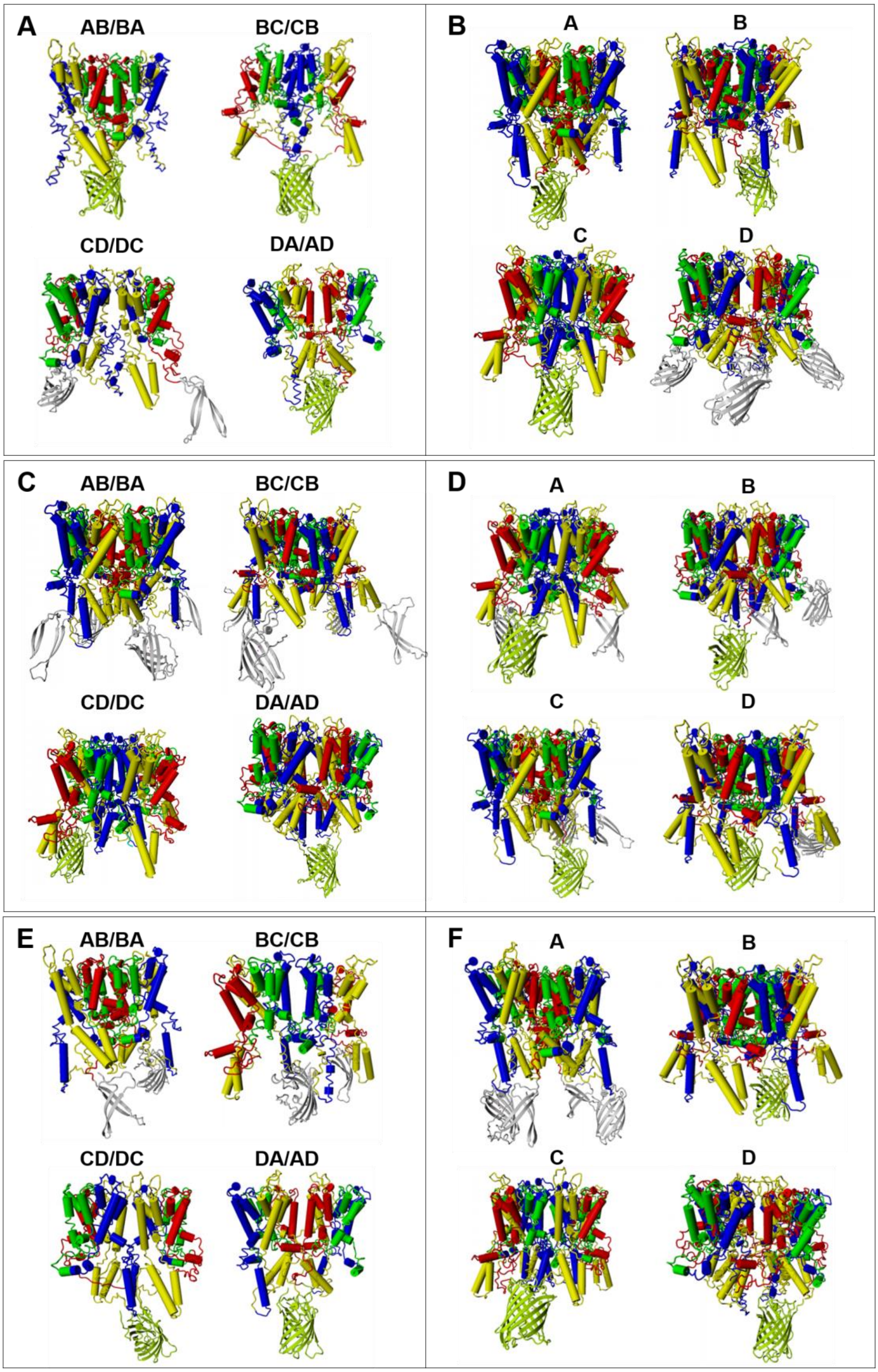

Supplementary Figure S3: Possible BiFC with VN and VC fused to N- and C-termini of Trk1[ΔLHL] monomers and present in different combinations. **A**: VN/Trk1[ΔLHL] with VC/Trk1[ΔLHL] - dimers - note that BiFC in the BC/CB and DA/AD dimers could not be predicted unambiguously because it might be prevented by steric hindrance (clashes), **B**: same - tetramers; **C**: VN/Trk1[ΔLHL] with Trk1[ΔLHL]/VC dimers, **D**: -tetramers; **E**: VC/Trk1[ΔLHL] with Trk1[ΔLHL]/VN - dimers, **F**: - tetramers.

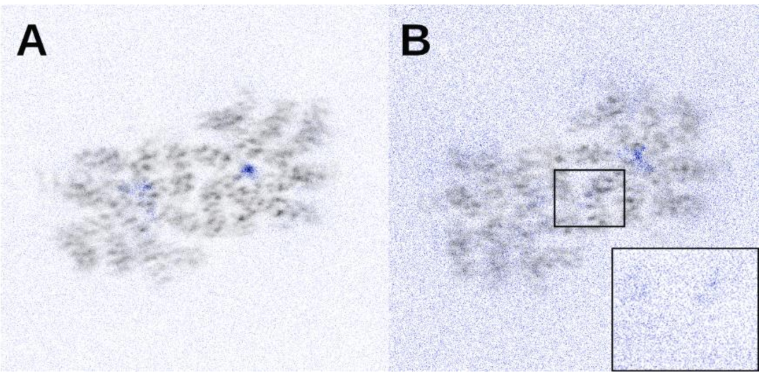

Supplementary Figure S4: Analysis of the Cl^- ion density during MD simulations of Trk1 (CD/DC dimer). Blurred blue dots represent Cl^- ions plot from all MD snapshots on XY plane. Grey colour is used to represent projection of protein C-α atoms. Data are collected from two independent MD runs (100 ns – **A**; 200 ns **B**). A Small increase of Cl^- density in the inter-domain space was seen in the second simulation and is shown in the inset.

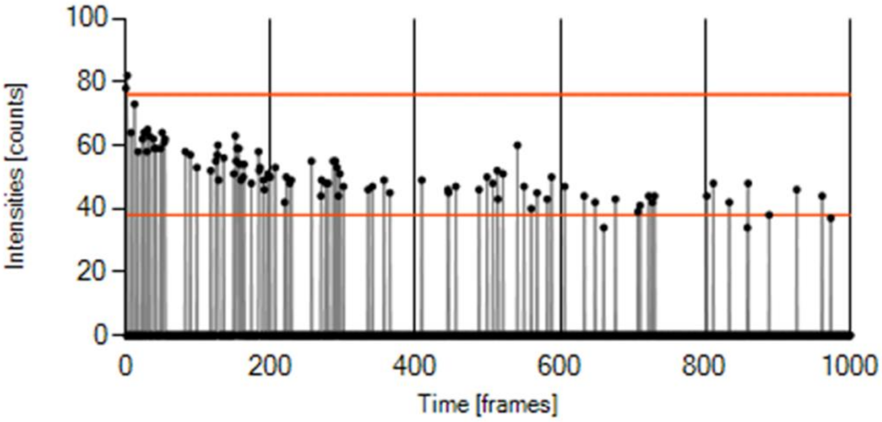

Supplementary Figure S5: Typical stepwise photobleaching trace of Trk1/GFP inside the cell. The data was processed as described in Supplementary Protocol “Analysis of stepwise photobleaching of GFP in cells".

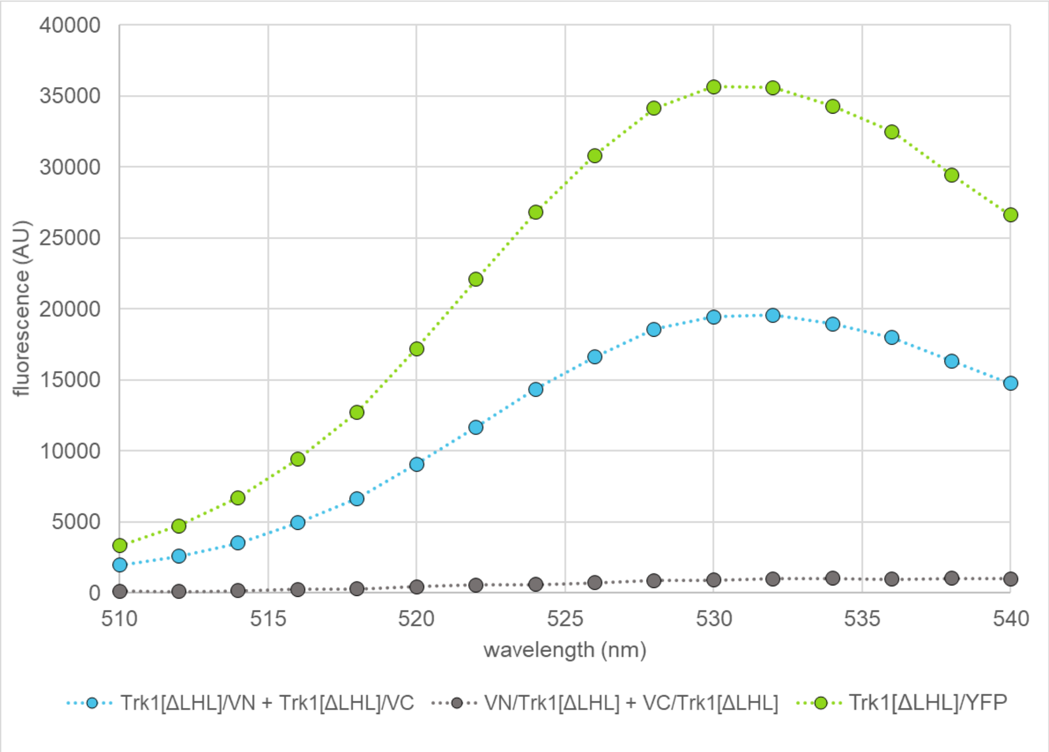

Supplementary Figure S6: BiFC fluorescence spectra of yeast cells grown in medium with 1 mM KCl possessing the indicated Trk1[ΔLHL] fusion proteins. Spectra were recorded in stationary phase (OD ~ 10; growth time: ~ 4 d).

- ***Supplementary Table S1:*** Episomal plasmids for expression of Trk1([ΔLHL]) fusion constructs

**Short name**	**Full name**	**Marker**	**Polypeptide composition**	**Reference**
Trk1[ΔLHL]/YFP-L	pYEX-[*LEU2*]-*TRK1[ΔLHL]/YFP*	*LEU2,URA3*	**TΔ**/YFP	This study
Trk1/VN-L	pYEX-[*LEU2*]-*TRK1/VN*	*LEU2,URA3*	**Trk1**/RSIAT/**VN**	(Kale *et al.*, 2019)
Trk1/VC-H	pYEX-[*HIS3*]-*TRK1/VC*	*HIS3,URA3*	**Trk1**/RPACKIPNDLKQKVMNH/**VC**	(Kale *et al.*, 2019)
Trk1[ΔLHL]/VN-L	pYEX-[*LEU2*]-*TRK1[ΔLHL]/VN*	*LEU2,URA3*	**TΔ**/RSIAT/**VN**	(Kale *et al.*, 2019)
Trk1[ΔLHL]/VC-H	pYEX-[*HIS3*]-*TRK1[ΔLHL]/VC*	*HIS3,URA3*	**TΔ**/RPACKIPNDLKQKVMNH/**VC**	(Kale *et al.*, 2019)
Trk1[ΔLHL]/VN-H	pYEX-[*HIS3*]-*TRK1[ΔLHL]/VN*	*HIS3,URA3*	**TΔ**/RSIAT/**VN**	This study
Trk1[ΔLHL]/VC-L	pYEX-[*LEU2*]-*TRK1[ΔLHL]/VC*	*LEU2,URA3*	**TΔ**/RPACKIPNDLKQKVMNH/**VC**	This study
VN/Trk1[ΔLHL]-H	pYEX-[*HIS3*]-*VN/TRK1[ΔLHL]*	*HIS3,URA3*	**VN**/RSIAT/**TΔ**	This study
VC/Trk1[ΔLHL]-L	pYEX-[*LEU2*]-*VC/TRK1[ΔLHL]*	*LEU2,URA3*	**VC**/RSIAT/**TΔ**	This study
VN/Trk1[ΔLHL]-L	pYEX-[*LEU2*]-*VN/TRK1[ΔLHL]*	*LEU2,URA3*	**VN**/RSIAT/**TΔ**	This study
VN/Trk1[ΔLHL]/VC-L	pYEX-[*LEU2*]-*VN/TRK1[ΔLHL]/VC*	*LEU2,URA3*	**VN**/RSIAT/**TΔ**/RPACKIPNDLKQKVMNH/**VC**	This study
Trk1[ΔLHL][G1010/GC/E1011]-H	pYEX-[*HIS3*]-*TRK1[ΔLHL][G1010/GC/E1011]*	*HIS3,URA3*	**Trk1**(M1-A163/G735-G1010)/AGGGAG/**GC**/GG/**Trk1**(E1011-L1235)	This study

Trk1[ΔLHL] - **TΔ**: Trk1 (M1-A163/G735-L1235); **GC**: yEGFP: D155-K238; **YFP**: M1-K238 **VN**: Venus M1-A155; **VC**: Venus D156-K239; linker sequences are given in one letter code.

Supplementary Table S2: Yeast strains generated by (co-) transformation of BY4741 *trk1,trk2,tok1Δ* (BYT123) with episomal plasmids for production of Trk1([ΔLHL]) fusion constructs.

**Yeast strain short name**	**Plasmids used to (co-)transform BY4741- *trk1,trk2,tok1***	**Marker**
Trk1[ΔLHL]/YFP	Trk1[ΔLHL]/YFP-L	*LEU2,URA3*
Trk1[ΔLHL]/VN	Trk1[ΔLHL]/VN-L	*LEU2,URA3*
Trk1[ΔLHL]/VC	Trk1[ΔLHL]/VC-L	*LEU2,URA3*
VN/Trk1[ΔLHL]/VC	VN/Trk1[ΔLHL]/VC-L	*LEU2,URA3*
Trk1/VN + Trk1/VC	Trk1/VN-L and Trk1/VC-H	*HIS3, LEU2,URA3*
Trk1[ΔLHL]/VC + Trk1[ΔLHL]/VN	Trk1[ΔLHL]/VC-L and Trk1[ΔLHL]/VN-H	*HIS3, LEU2,URA3*
VN/Trk1[ΔLHL] + Trk1[ΔLHL]/VC	VN/Trk1[ΔLHL] and Trk1[ΔLHL]/VC-H	*HIS3, LEU2,URA3*
VN/Trk1[ΔLHL] + VC/Trk1[ΔLHL]	VN/Trk1[ΔLHL]-H and VC/Trk1[ΔLHL]-L	*HIS3, LEU2,URA3*
Trk1[ΔLHL][G1010/GC/E1011] + Trk1[ΔLHL]/VN	Trk1[ΔLHL][G1010/GC/E1011]-H and Trk1[ΔLHL]/VN-L	*HIS3, LEU2,URA3*

Supplementary Protocol: Analysis of stepwise photobleaching of GFP in cells

In the case of single GFP signals in cells, first cell segmentation is performed. The segmentation is done via a U-net model (Falk *et al.*, 2019), which creates a mask based on the sequence's first image. Each pixel classified as a background has the value 0. Each pixel classified as part of a cell displays a value of 1. Hence, detected EVs can be filtered based on their position and the corresponding value in the mask.

Next, our platform quantifies stepwise photobleaching of fluorescent molecule signals (e.g., sparsely distributed GFP-labelled proteins). Due to the partially low intensities of the single emitters and illumination irregularities of the images, homogeneous thresholding techniques are not feasible. Instead, a kernel filter is used for signal identification by considering the size and brightness of the signals. The kernel consists of a window of a predefined size that scans over each image pixel-wise while classifying each pixel value as background or signal. The kernel is divided into 3 regions, foreground, sigma, and background.

The foreground describes the inner area of the kernel, which defines the fluorescence signal area. The background describes the area in the outer ring of the kernel, and the sigma describes the ring-shaped area in-between the fore- and background used for spacing. The foreground- and background signal intensities are determined by calculating the averaged pixels' intensities of the areas. Suppose the intensity difference between foreground and background is higher than a certain threshold and exceeds a minimum intensity. The current centre pixel is counted as a fluorescent signal in that case. The analysis determines a set of individual fluorescence signals in each image within an image sequence. Next, the connection of the signals between the images is established. Tracking starts on the first image for each determined fluorescence signal. A new track is started for each detected signal on the first image.

Starting from the second image, the spatial distance between each signal and the previous image's signals is computed. Suppose the distance between the closest two fluorescence signals in two following images is below a user-defined threshold. In that case, they are associated with each other. A fluorescence signal, which could not be associated with a pre-existing track, is regarded as a new track. Thus, a set of tracks is generated.

The signal intensity depends on the quantity of fluorescent emitters. Here, it is assumed that the signal of an average single emitter is constant (bleaching and inherent fluctuations disregarded). The intensities of multiple fluorescent molecules behave additively (quenching is disregarded), with their intensities only being influenced by noise and illumination irregularities. Due to photobleaching within the image sequence, a temporal, stepwise drop in the signal intensities is observable. Hence, analysing this drop enables the determination of the intensity of a single emitter and the number of emitters per fluorescence signal. Due to these drops, the observed intensities of a signal can be clustered based on their similarity. The equidistant cluster centres are sought for these clusters, which best describe the data. The intensity distance between clusters is equivalent to the intensity of a single emitter and is referred to as delta.

An exhaustive search is performed to determine the value of a single emitter from the time course of the photobleaching. In this search, with a step size of 1, all values starting from 1 up to the highest intensity of a tracked signal are set as delta. For each delta, equidistant centres are generated, and the individually tracked signal intensities are assigned to the closest centre. Finally, the quality of the resulting clusters is evaluated by the Davies Bouldin Index (DBI, a metric for the clustering quality).

The DBI is always between zero and infinite, with a lower number representing a better fit. The calculated value is based on the distance between the centres to their data points and the distance between the individual centres

(estimated single emitter signals). The DBI aims to minimize the distance between centres and their data points while maximizing the distance between individual clusters.

By evaluating the cluster fit with the DBI and choosing the delta with the lowest score, the best fitting centres for the signal's intensity data are determined. Thus, the intensity of a single emitter is equivalent to the found delta, and the number of centres determines the number of emitters.
